# Supplementary material for: Methane emissions from thermokarst lakes must emphasize the ice-melting impact on the Tibetan Plateau
Source: Nat Commun. 2025 Mar 11;16:2404. doi: 10.1038/s41467-025-57745-2 (PMC11894136; doi:10.1038/s41467-025-57745-2)
Supplement: Supplementary file 1 — Supplementary Information [file 41467_2025_57745_MOESM1_ESM.pdf]

Supplementary Information for

Methane emissions from thermokarst lakes must emphasize the ice-melting impact on the Tibetan Plateau

Cuicui Mu[1,2,3*], Pengsi Lei[1], Mei Mu[1], Chunling Zhang[1], Zhensong Zhou[1], Jinyue Song[1], Yunjie Jia[1], Chengyan Fan[1], Xiaoqing Peng[1], Guofei Zhang[1], Yuanhe Yang[4], Lei Wang[5], Dongfeng Li[6], Chunlin Song[7], Genxu Wang[7], Zhen Zhang[8]

[1]Key Laboratory of Western China's Environmental Systems (Ministry of Education), College of Earth and Environmental Sciences, Observation and research station on Eco-Environment of Frozen Ground in the Qilian Mountains, Lanzhou University; Lanzhou, China.

[2]State Key Laboratory of Frozen Soil Engineering, Northwest Institute of Eco-Environment and Resources, Chinese Academy of Sciences; Lanzhou, China.

[3] Qinghai–Beiluhe Plateau Frozen Soil Engineering Safety National Observation and Research Station, Lanzhou, China.

[4]State Key Laboratory of Vegetation and Environmental Change, Institute of Botany, Chinese Academy of Sciences, Beijing, China

[5]Advanced Interdisciplinary Institute of Environment and Ecology, Beijing Normal University; Zhuhai, China.

[6]Key Laboratory for Water and Sediment Sciences, Ministry of Education, College of Environmental Sciences and Engineering, Peking University; Beijing, China.

[7]State Key Laboratory of Hydraulics and Mountain River Engineering, College of Water Resource and Hydropower, Sichuan University; Chengdu, China.

[8]National Tibetan Plateau Data Center, State Key Laboratory of Tibetan Plateau Earth System, Environment and Resource, Institute of Tibetan Plateau Research, Chinese Academy of Sciences, Beijing, China.

*Corresponding author. Email: mucc@lzu.edu.cn.

Supplementary Table 1. Basic information of the investigated thermokarst lakes during the ice-covered period on the Tibetan Plateau. The vegetation types include alpine swamp meadow (ASM), alpine meadow (AM), alpine steppe (AS) and alpine desert (AD).

Name of thermokarst lakes	Long (°E)	Lat (°N)	Area (km^2)	Altitude (m)	Vegetation types
AD42	91.87	32.68	0.1445	4988	AM
AD43	91.87	32.68	0.0879	4989	AM
AD44	91.86	32.57	0.0058	5127	ASM
AD45	91.86	32.57	0.0011	5127	ASM
AD54	91.87	32.59	0.0046	5085	AM
ADa	91.87	32.67	0.0560	4994	AM
ADb	91.87	32.67	0.0801	4995	AM
ADc	91.87	32.67	0.0070	4990	AM
ADd	91.87	32.67	0.0081	4995	AM
ADe	91.86	32.66	0.0303	4995	AM
B1	92.89	34.83	0.0067	4656	ASM
B11	92.92	34.83	0.0029	4647	ASM
B12	92.92	34.83	0.0023	4647	ASM
B15	92.92	34.83	0.0134	4638	AM
B16	92.93	34.83	0.0026	4637	AM
B2	92.90	34.83	0.0015	4656	ASM
B3	92.90	34.83	0.0009	4658	AM
B4	92.90	34.83	0.0004	4658	AM
B5	92.90	34.83	0.0004	4662	AM
B7	92.90	34.83	0.0013	4656	ASM
B8	92.90	34.82	0.0012	4657	ASM
B9	92.90	34.82	0.0019	4655	AM
Ba	92.90	34.83	0.0007	4655	ASM
Bb	92.90	34.83	0.0010	4660	AM
Bc	92.90	34.83	0.0008	4658	AM
Bd	92.90	34.83	0.0008	4662	AM
BDQ10	94.08	35.06	0.0221	4529	AM
BDQ2	93.87	35.52	0.0144	4618	AS
BDQ3	93.90	35.32	0.0153	4466	AD
BDQ4	93.90	35.24	0.0787	4421	AD
BDQ5	93.93	35.23	0.0421	4418	AD
BDQ6	93.94	35.20	0.6490	4414	AM
BDQ7	93.95	35.18	0.0226	4426	AM
BDQ8	94.00	35.10	0.0220	4480	ASM
BDQ9	94.08	35.06	0.0515	4527	AM
C1	93.33	35.33	0.0014	4536	AD
C3	93.02	35.06	0.0692	4645	AD
C4	93.44	35.37	0.0084	4502	AM
MD10	98.39	34.83	0.0083	4214	AM

MD11	98.39	34.83	0.0004	4215	AM
MD26	98.11	34.74	0.0147	4232	AM
MD7	98.33	34.83	0.0576	4210	AD
MDa	98.51	34.92	0.0364	4244	AS
MDb	98.11	34.74	0.0029	4231	AS
T3	92.47	34.25	0.0043	4548	AS
T4	92.47	34.25	0.0043	4554	AS
T7	92.47	34.25	0.0040	4551	AS
T8	92.49	34.28	0.0059	4560	AS
T9	92.46	34.23	0.0399	4540	AS
Ta	92.45	34.23	0.0150	4542	AS
WDL1	93.08	35.20	0.0106	4632	AS
WDL6	93.62	35.45	0.0054	4469	AD
WDLa	93.08	35.20	0.0127	4635	AS
WDLb	93.59	35.43	0.0291	4465	AD
WS2	93.08	35.20	0.0008	4628	AM
X2	92.96	34.96	0.0312	4559	AD

31

Supplementary Table 2. Relationships of dissolved CH_4 concentrations between the ice-covered and ice-melting periods. In the equations, Y means the CH_4 concentrations during the ice-covered period, and X means the CH_4 concentrations during the ice-free period.

Vegetation types	Equations	R^2	P
**Alpine swamp meadow**	Y= 10.69X + 14.88	0.64	< 0.05
**Alpine meadow**	Y= 18.94X + 3.70	0.66	< 0.001
**Alpine steppe**	Y= 1.27X + 0.59	0.70	< 0.05
**Alpine desert**	Y= 0.92X + 0.02	0.83	< 0.001

Supplementary Table 3. Environmental factors influencing the susceptibility of thermokarst lakes. These factors include topography, hydrology, soil, human activities, permafrost and climate.

Environmental Factors		Resolution	Sources	Website
Topography	Elevation, Slope	1 km	National Centers for Environmental Information	https://www.ncei.noaa.gov/
Hydrology	Topographic wetness index			
Soil	Silt, Sand	250 m	Harmonized World Soil Database version 2.0	https://doi.org/10.4060/cc3823en
Permafrost	Active layer thickness		Peng et al., 2023[1]	
Human activity	Human footprint Index	1 km	Last of the Wild, v2	https://sedac.ciesin.columbia.edu/
Climate	Rainfall, Temperature	1 km	WorldClim	https://www.worldclim.org/

Supplementary Table 4. The accuracy evaluation of machine learning Models. The susceptibility of thermokarst lakes is simulated using six machine learning models including Random Forest (RF), Generalized Additive Model (GAM), Generalized Boosted Regression Model (GBM), Classification and Regression Tree Analysis (CTA), Artificial Neural Network (ANN), and MaxEnt. We evaluate the models' performance using Receiver Operating Characteristic (ROC) curves, Kappa statistics (KAPPA), and True Skill Statistics (TSS).

Models	AUC	KAPPA	TSS
RF	0.963	0.822	0.823
CTA	0.905	0.719	0.718
GAM	0.923	0.689	0.690
GBM	0.924	0.688	0.689
MAXENT	0.890	0.618	0.620

Supplementary Table 5. Area density of thermokarst lakes under different vegetation types. Area density refers to the area of lakes within a 1 pixel. We calculate the average area density with moderate to very high susceptibility of thermokarst lakes for different vegetation types.

**Vegetation types**	**Pixel count**	**Thermokarst lake density (m^2/pixel)**	**Standard Error (m^2/km^2)**
Alpine swamp meadow	27838	4406.47	148.30
Alpine meadow	118477	6160.54	145.05
Alpine steppe	104321	4833.68	152.42
Alpine desert	108139	5677.58	158.42

Supplementary Table 6. Projected CH_4 emissions from thermokarst lakes on the Tibetan Plateau under SSP scenarios during ice-free and ice-melting periods by 2050 and 2100. Data are presented as mean ± standard error (SE).

Periods		CH_4 emissions (Gg C yr^{-1})			
		Ice-melting period		Ice-free period	
		No lake ice change	Lake ice loss	No lake ice change	Lake ice loss
1969	Luo et al., 2023[2]	5.93	6.39	28.71	26.96
2020	This study	11.21 ± 1.64		54.24 ± 9.90	
	SSP1-2.6	19.83 ± 5.17	18.92 ± 4.93	96.99 ± 23.44	100.46 ± 24.28
2050	SSP2-4.5	19.93 ± 5.19	19.01 ± 4.95	97.42 ± 23.55	100.90 ± 24.39
	SSP5-8.5	19.80 ± 5.16	18.89 ± 4.93	96.86 ± 23.41	100.32 ± 24.25
	SSP1-2.6	21.72 ± 5.65	19.06 ± 4.95	106.27 ± 25.74	116.41 ± 28.20
2100	SSP2-4.5	21.88 ± 5.69	19.20 ± 4.99	107.04 ± 25.93	117.25 ± 28.40
	SSP5-8.5	23.06 ± 5.99	20.23 ± 5.25	112.79 ± 27.34	123.55 ± 29.95

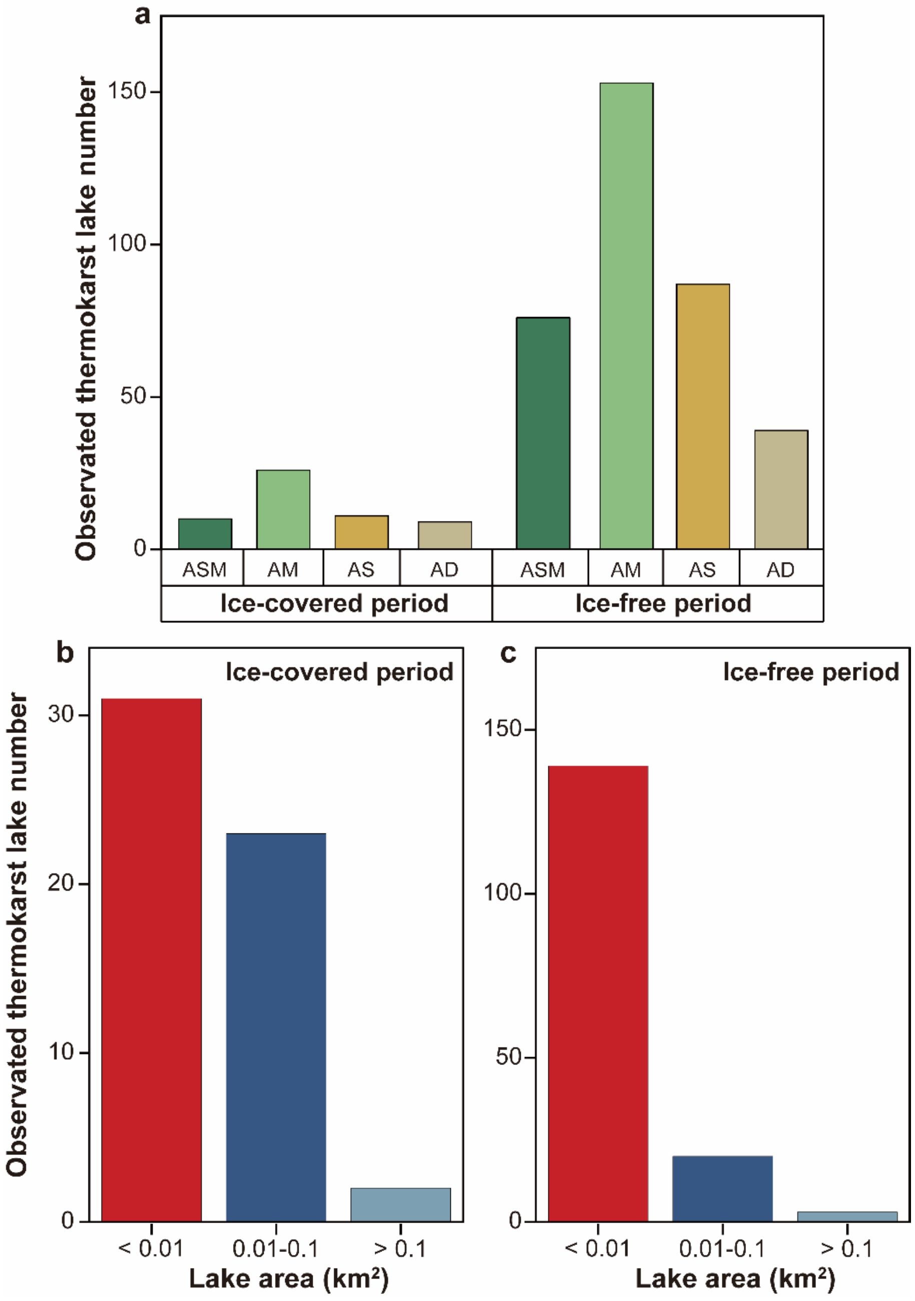

Supplementary Figure 1. The number of observated thermokarst lakes with different vegetation types and lake areas. (**a**) The diagrams show the number distribution of measured thermokarst lakes under four vegetation types: alpine swamp meadow (ASM), alpine meadow (AM), alpine steppe (AS), and alpine desert (AD)**.** The diagrams show the number distribution of observed thermokarst lakes with different areas during the ice-covered (**b**) and ice-free periods (**c**).

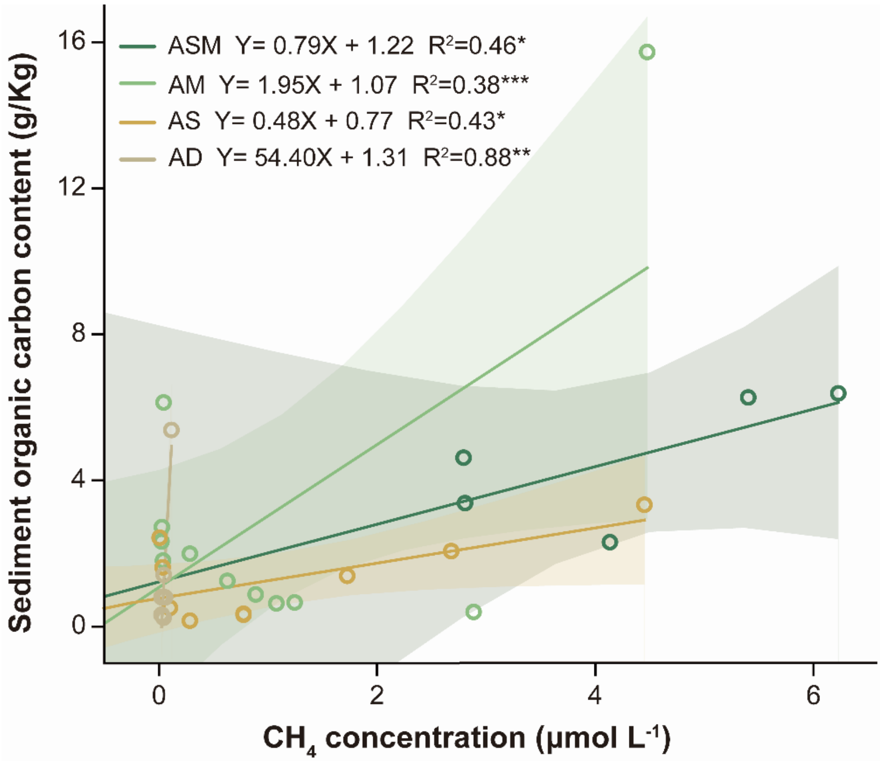

Supplementary Figure 2. The correlation between dissolved CH_4 concentrations and sediment organic carbon contents in thermokarst lakes. Samples were collected during the ice-free periods from 2019 to 2023 at Beilu River, Wudao Liang, and Tanggula[3]. The shaded area represents the 95% confidence interval. *P < 0.05, **P < 0.01, ***P < 0.001.

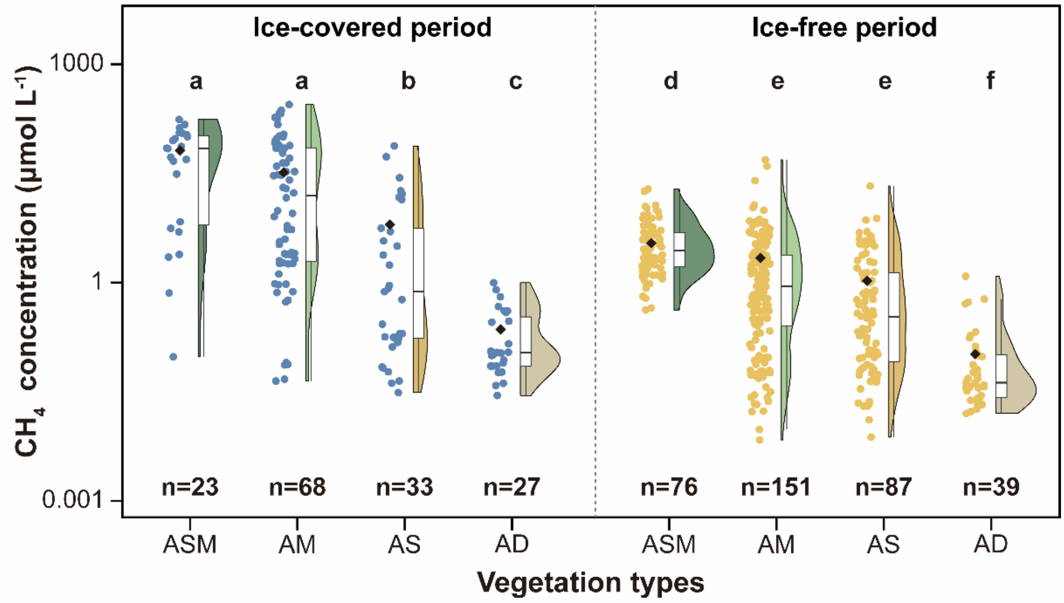

Supplementary Figure 3. Comparison of dissolved CH_4 concentrations between the ice-covered and ice-free periods. The diagram shows the pattens of dissolved CH_4 concentrations in both ice-covered and ice-free periods with surrounding vegetation types. The violin plot illustrates the magnitude and distribution density of CH_4 concentrations of individual samples, in which the boxes represent the 25th and 75th percentiles, the black line indicates the median value, and the black diamond represents the mean value. The letters of a-f represent the significant differences. We used one-way analysis of variance (ANOVA) with Tukey's HSD post hoc comparisons to test differences in concentrations across different vegetation types and periods at a significance level of $P < 0.05$.

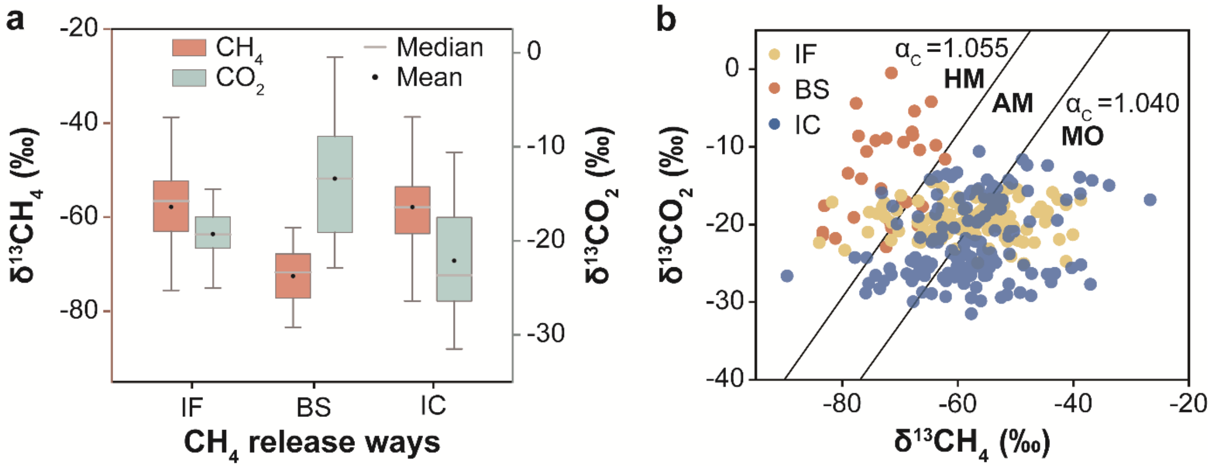

Supplementary Figure 4. Stable carbon isotopes of dissolved CH_4 and CO_2 in thermokarst lakes on the Tibetan Plateau. (**a**) Comparison of $\delta^{13}CH_4$ and $\delta^{13}CO_2$ in different pathways the CH_4 release through. IC means the CH_4 released by diffusion during the ice-covered period. IF means the CH_4 release through diffusion during the ice-free period[3], and BS means CH_4 release through ebullition[4]**.** (**b**) The relationship of $\delta^{13}CH_4$ and $\delta^{13}CO_2$ in thermokarst lakes. The black lines are the apparent carbon fractionation factor (α_C) values of 1.040 and 1.055. The α_C values indicate the production pathway or oxidation state of CH_4. $\alpha_C > 1.055$ suggests that CH_4 is mainly produced by CO_2 reduction (hydrogenotrophic methanogenesis, HM). $1.040 < \alpha_C < 1.055$ suggests that CH_4 is produced by acetate fermentation (acetoclastic methanogenesis, AM). When α_C is less than 1.040, it shows that CH_4 oxidation is the dominant process in thermokarst lakes (methane oxidation, MO).

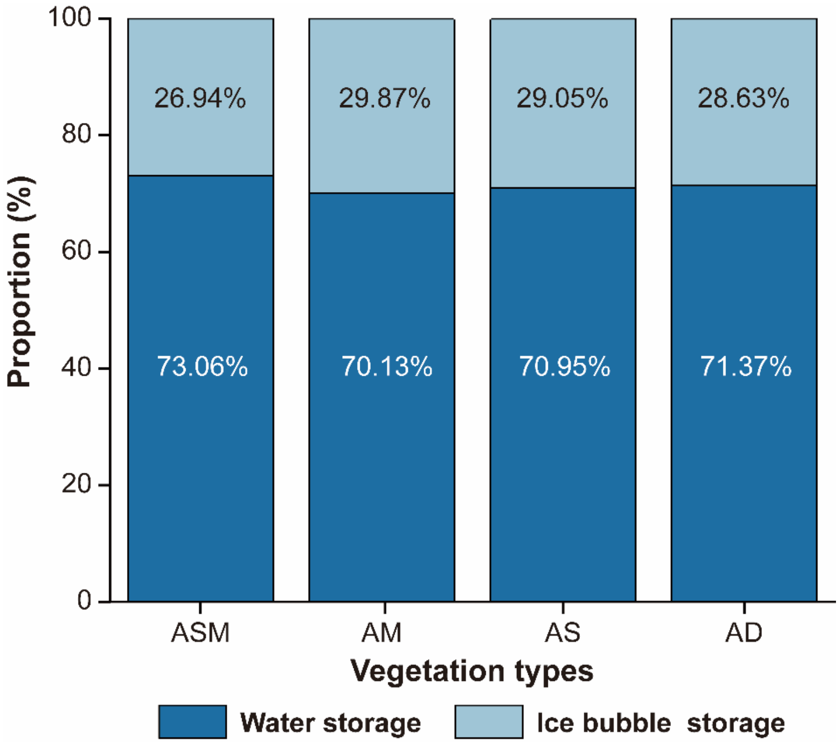

Supplementary Figure 5. The proportion of CH_4 emissions from water storage and ice bubble storage from thermokarst lakes during ice-melting period.

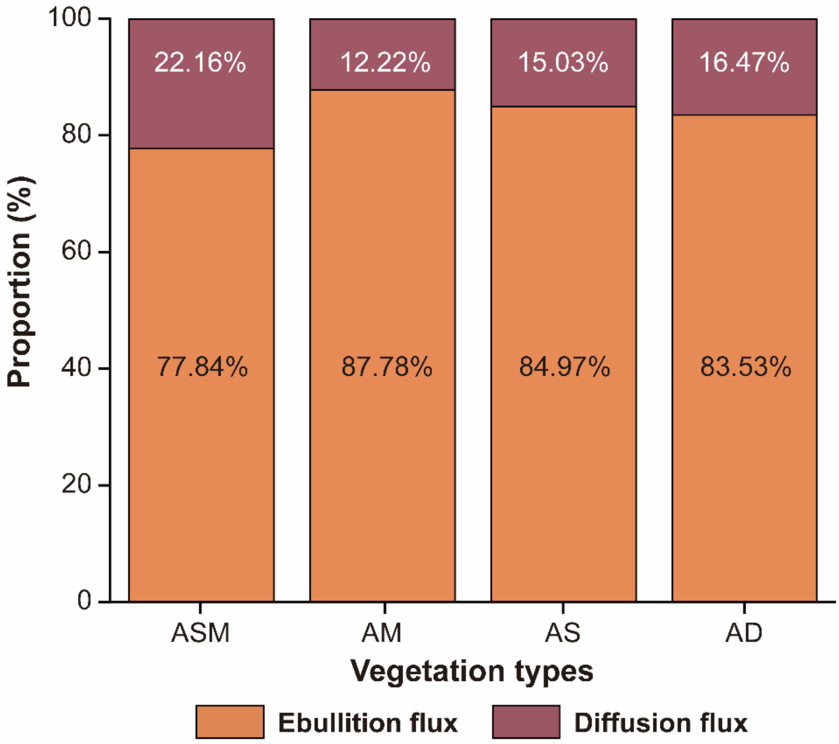

Supplementary Figure 6. The proportion of CH_4 emissions through ebullition and diffusion from thermokarst lakes under different vegetation types. The data was obtained from Yang et al. (2023)[4].

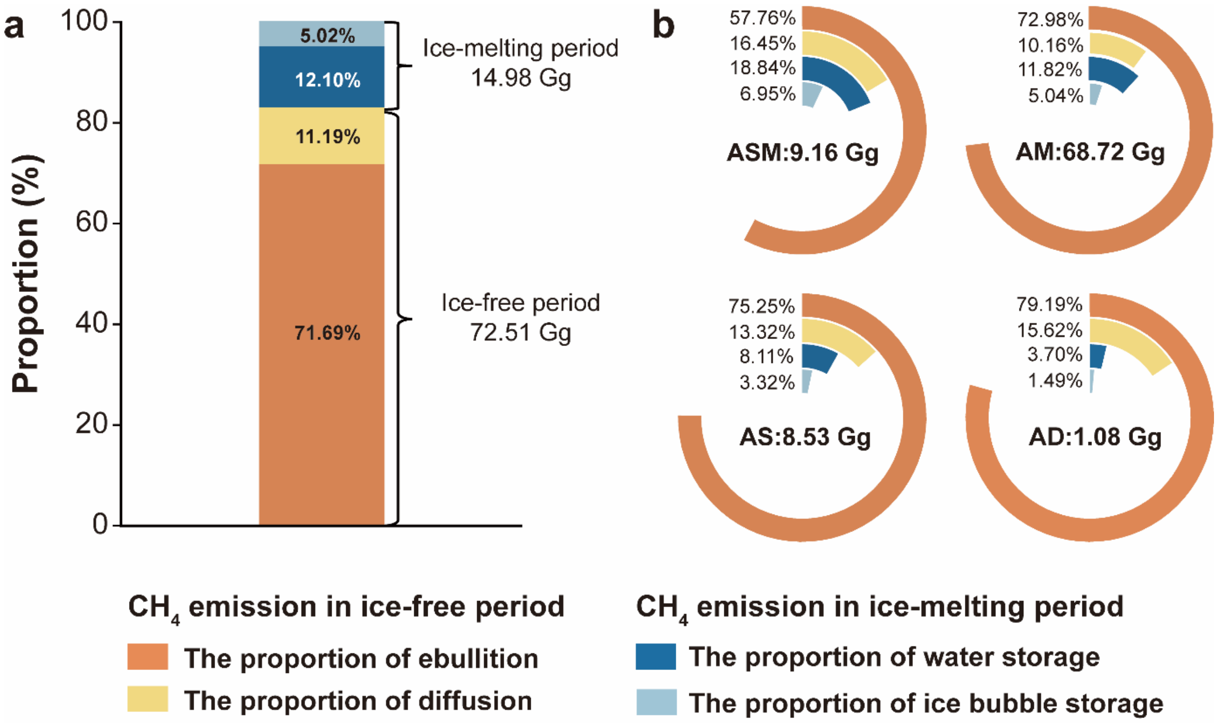

Supplementary Figure 7. The estimated CH_4 emissions from thermokarst lakes on the Tibetan Plateau. (**a**) Proportions of CH_4 emissions from water storage, ice bubble storage, ebullition and diffusion in annual total CH_4 emissions. (**b**) Proportions of CH_4 emissions in different ways during ice-melting and ice-free periods under alpine swamp meadow (ASM), alpine meadow (AM), alpine steppe (AS) and alpine desert (AD) The number at centers of the circle indicates the annual CH_4 emissions from thermokarst lakes with the surrounding vegetation type (Gg CH_4 yr^{-1}).

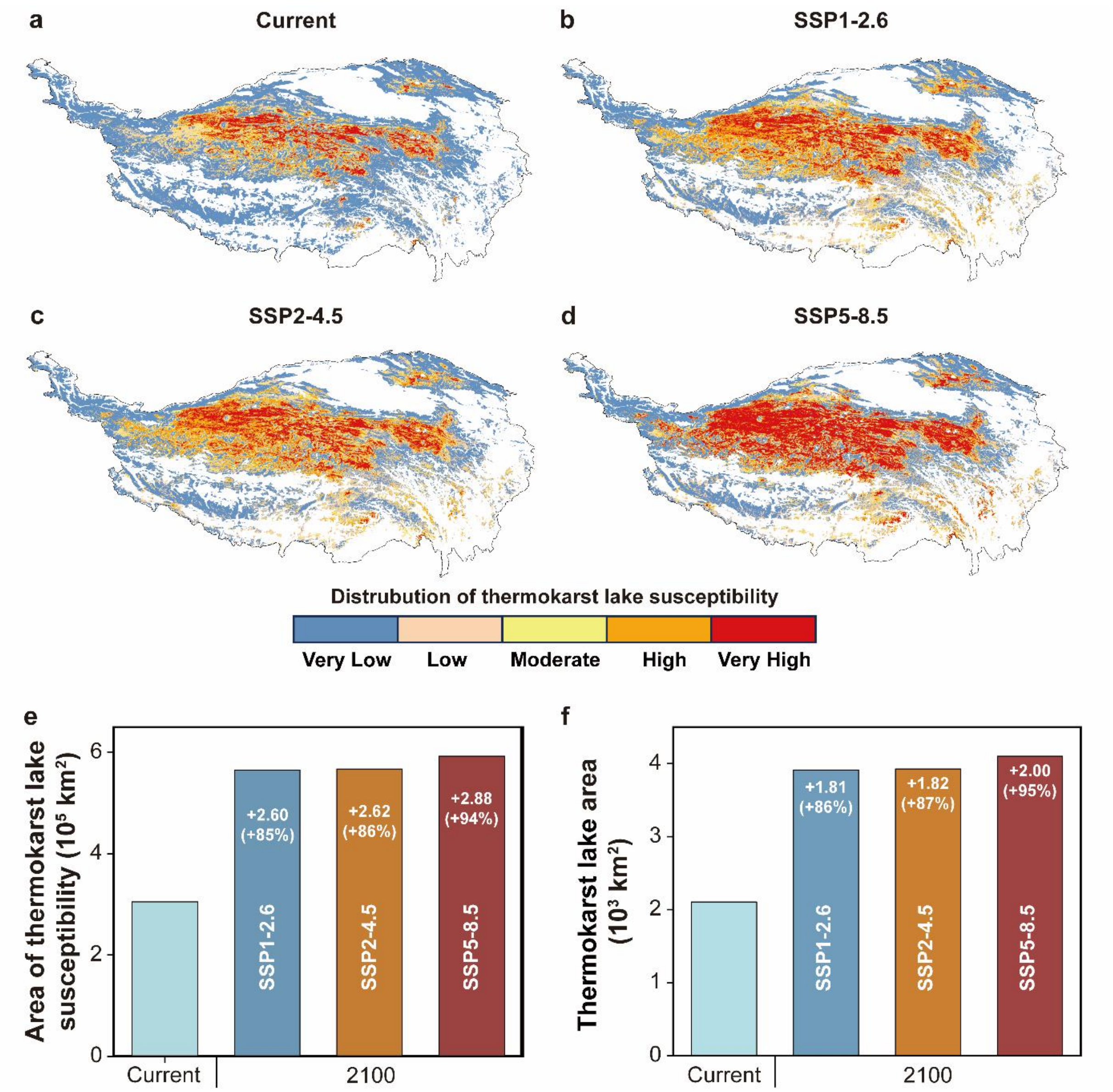

Supplementary Figure 8. Projected thermokarst lakes susceptibility on the Tibetan Plateau. Current **(a)** and future **(b-d)** distribution of thermokarst lake susceptibility with five levels of very low, low, moderate, high and very high susceptibility on the Tibetan Plateau. (**e**) The total areas of thermokarst lake susceptibility with moderate, high, and very high levels, identified as thermokarst lake-affected areas, are considered for both the current period and the year 2100. (**f**) Predictions for future expansion of thermokarst lakes.

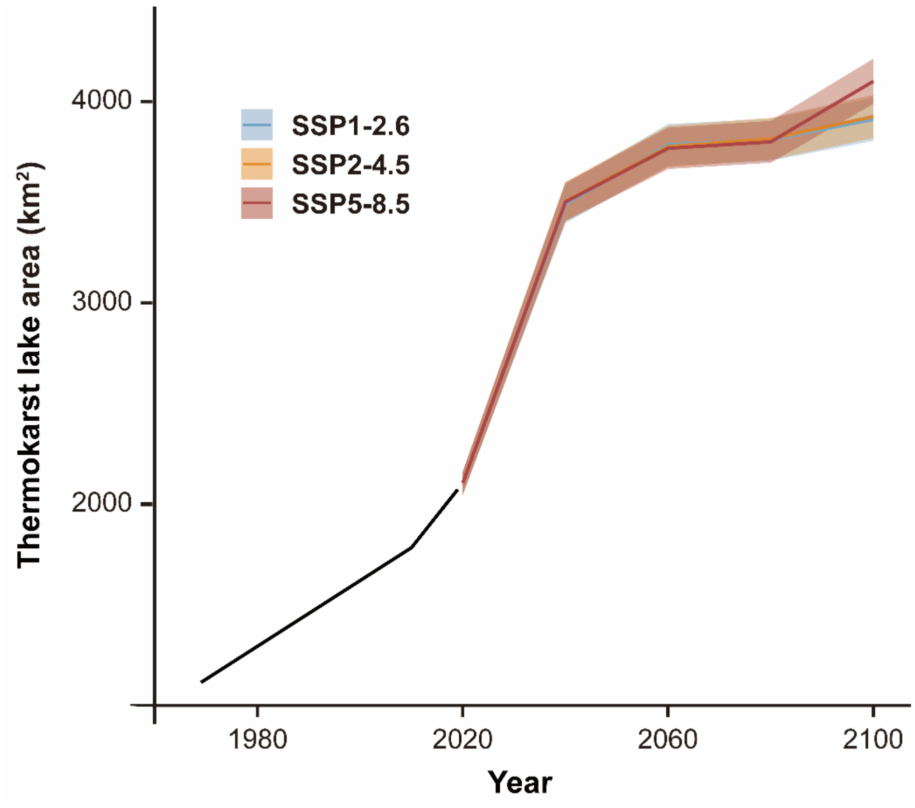

Supplementary Figure 9. Changes in the area of thermokarst lakes on the Tibetan Plateau from 1969 to 2100. The area and change rate of thermokarst lakes on the Tibetan Plateau from 1969 to 2019 are derived from the previous study[2]. The shaded area represents the error range (SE).

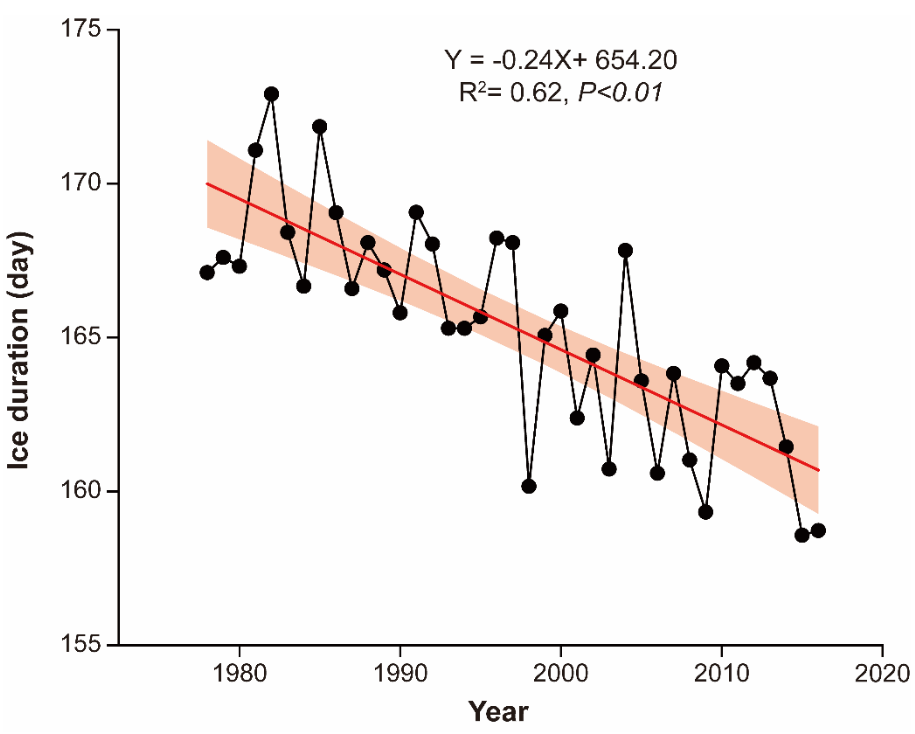

Supplementary Figure 10. Changes of mean lake ice duration on the Tibetan Plateau from 1978 to 2016. The average ice duration on the Tibetan Plateau has decreased at the rate of 0.2 d yr^{-1}. We utilized the annual average ice duration data from a lake ice phenology dataset[5], which covers 132 lakes on the Tibetan Plateau. The shaded area represents the 95% confidence interval.

References

1 Peng, X. *et al.* Active layer thickness and permafrost area projections for the 21st century. *Earth's Future* **11**, e2023EF003573 (2023).

2 Luo, J. *et al.* Abrupt increase in thermokarst lakes on the central Tibetan Plateau over the last 50 years. *Catena* **217**, 106497 (2022).

3 Mu, C. *et al.* High carbon emissions from thermokarst lakes and their determinants in the Tibet Plateau. *Global Change Biology* **29**, 2732-2745 (2023).

4 Yang, G. *et al.* Characteristics of methane emissions from alpine thermokarst lakes on the Tibetan Plateau. *Nature Communications* **14**, 3121 (2023).

5 Guo, L. *et al.* in *Lake ice phenology dataset across the Tibetan Plateau during 1978-2016* (ed Center National Tibetan Plateau Data) (2022).
